# Supplementary material for: Mixed Dye Removal Efficiency of Electrospun Polyacrylonitrile–Graphene Oxide Composite Membranes
Source: Polymers (Basel). 2020 Sep 3;12(9):2009. doi: 10.3390/polym12092009 (PMC7563693; doi:10.3390/polym12092009)
Supplement: Supplementary file 1 [file polymers-12-02009-s001.pdf]

polymers

Supplementary Materials

Mixed Dye Removal Efficiency of Electrospun Polyacrylonitrile–Graphene Oxide Composite Membranes

Wongi Jang [1,2,†], Jaehan Yun [1,2,†], Younggee Seo [2], Hongsik Byun [2], Jian Hou [3,*] and Jun-Hyun Kim[1,*]

[1] Department of Chemistry, Illinois State University, Normal, Illinois 61790-4160, USA; wjang12@ilstu.edu (W.J.), jyun12@ilstu.edu (J.Y.)
[2] Department of Chemical Engineering, Keimyung University, Daegu 42601, South Korea; ygseo93@naver.com (Y.S.), hsbyun@kmu.ac.kr (H.B.)
[3] Department of Chemical Engineering, Zibo Vocational Institute, Zibo 255314, P. R. China
* Correspondence: houjimmy@naver.com (J.H.) and jkim5@ilstu.edu (J.-H.K.)
† These authors equally contributed to this work

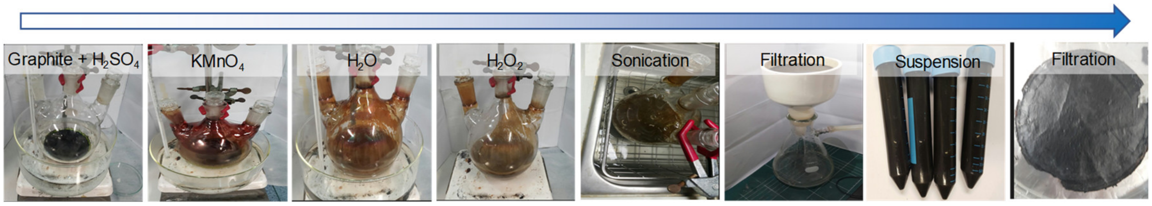

Figure S1. Preparation of GO via a modified Hummers' method.

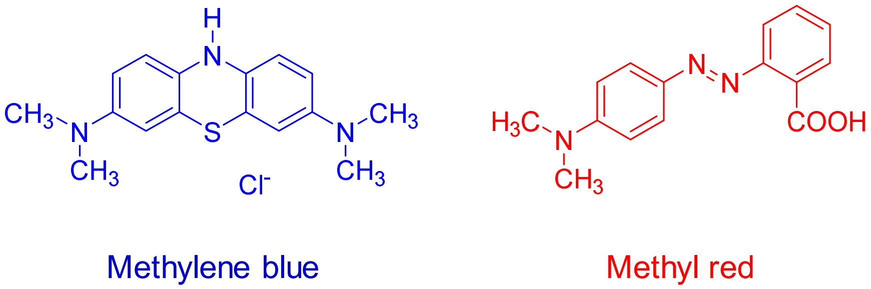

Figure S2. Structures of methylene blue (MB) and methyl red (MR).

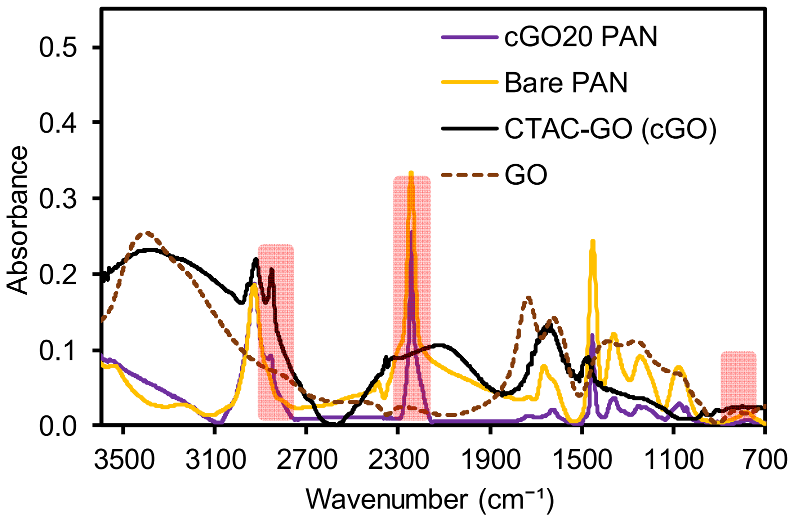

Figure S3. FT-IR spectra of GO, CTAC-modified GO (cGO), bare PAN, and cGO20 PAN composite nanofiber membranes (red highlights indicate the successful modification of GO with CTAC and their successful integration into the PAN nanofibers).

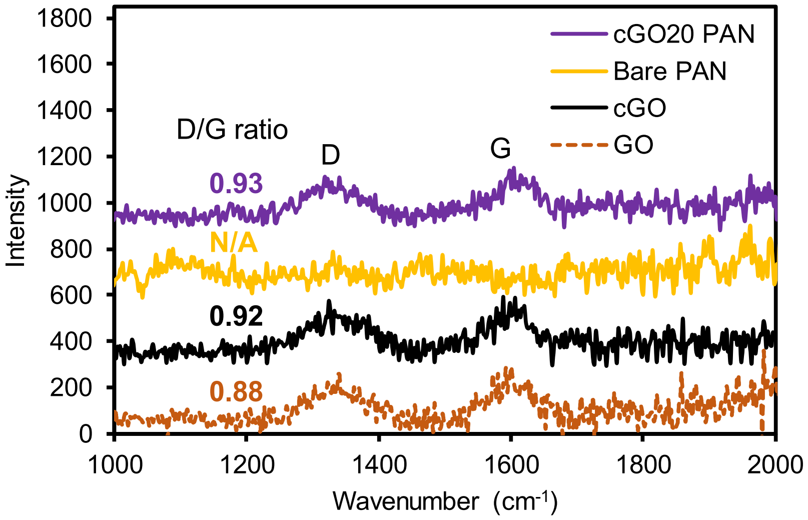

Figure S4. Raman spectra of GO, CTAC-modified GO (cGO), bare PAN, and cGO20 PAN composite nanofiber membrane.
